# Beyond computational predictions: toward mechanistic validation and systemic profiling of PFOA carcinogenicity

**DOI:** 10.1097/JS9.0000000000003116

**Published:** 2025-08-01

**Authors:** Lingling Xie, Tingting Chen

**Affiliations:** a The First School of Clinical Medicine, Zhejiang Chinese Medical University, Hangzhou, Zhejiang, China; b Zhejiang Key Laboratory of Integrative Chinese and Western Medicine for Diagnosis and Treatment of Circulatory Diseases, Zhejiang Hospital (Affiliated Zhejiang Hospital, Zhejiang University School of Medicine), Hangzhou, Zhejiang, China; c Zhejiang Engineering Research Center for Precise Diagnosis and Innovative Traditional Chinese Medicine for Cardiovascular Diseases, Zhejiang Hospital (Affiliated Zhejiang Hospital, Zhejiang University School of Medicine), Hangzhou, Zhejiang, China

*Dear Editor,*

Recently, we read with great interest in an article about the relationship between PFOA (perfluorooctanoic acid) and clear cell renal cell carcinoma (ccRCC)^[1]^. This study used network toxicology and molecular docking methods to find that PFOA may affect ccRCC through 70 potential targets and 7 key genes, such as CYP2C9 and CYP3A4, involving four mechanisms: abnormal xenobiotic metabolism, lipid homeostasis disorder, oxidative stress, and steroid hormone signaling pathway disorder. Molecular docking shows that PFOA binds tightly to these gene proteins. We have been assured that the article complies with TITAN Guidelines 2025-governing declaration and use of AI^[2]^.

While this work advances our understanding of PFOA’s role in clear cell renal cell carcinoma (ccRCC), several limitations warrant consideration. Firstly, the functional dynamics of key genes (e.g., CYP2C9, CYP3A4) across cancer progression stages remain undefined. For example, the polymorphism of CYP2C9 may affect the metabolism of various drugs, including anticoagulants and nonsteroidal anti-inflammatory drugs, which are commonly used for supportive treatment in cancer patients^[3]^, while CYP3A enzymes play dual roles in the activation and detoxification of carcinogens^[4]^—this aspect was not explored in this study. Secondly, although molecular docking confirmed the binding affinity of PFOA to its targets, its direct impact on carcinogenic pathways still needs to be verified through experiments^[5]^. Computational evidence alone cannot substantiate causality; orthogonal assays (e.g., CRISPR-based gene perturbation or dose-response studies) are essential to delineate mechanistic links. Finally, the exclusive focus on ccRCC limits generalizability. Epidemiologic data ties PFOA exposure to kidney cancer (primarily ccRCC)^[6]^, and its Group 1 carcinogen designation by IARC underscores global relevance. However, its effects on papillary/chromophobe RCC subtypes or extra-renal malignancies remain unexplored.

To address critical knowledge gaps, future research priorities include: (1) conducting longitudinal mechanistic investigations to profile temporal dynamics of CYP2C9 and CYP3A4 interactions across tumor progression stages, identifying stage-specific therapeutic vulnerabilities; (2) integrating computational predictions with functional validation using advanced models (e.g., 3D tumor organoids) to quantify PFOA-gene interactions and downstream oncogenic effects; and (3) expanding oncologic profiling to elucidate PFOA’s roles in non-ccRCC renal subtypes and extrarenal malignancies with established PFOA associations (e.g., testicular/hepatic carcinomas). These initiatives will delineate PFOA’s systemic carcinogenicity and advance targeted interventions for pollution-associated cancers.

In summary, this study provides important insights into the molecular mechanisms by which PFOA induces ccRCC, but future research should delve deeper into the dynamics of gene functions, experimental validation, and extend to other cancer types to better assess the carcinogenic potential of PFOA.

## Data Availability

Not applicable.
